# Implications of indoor microbial ecology and evolution on antibiotic resistance

**DOI:** 10.1038/s41370-019-0171-0

**Published:** 2019-10-07

**Authors:** Sarah Ben Maamar, Jinglin Hu, Erica M. Hartmann

**Affiliations:** Department of Civil and Environmental Engineering, Northwestern University, Evanston, IL USA

## Abstract

The indoor environment is an important source of microbial exposures for its human occupants. While we naturally want to favor positive health outcomes, built environment design and operation may counter-intuitively favor negative health outcomes, particularly with regard to antibiotic resistance. Indoor environments contain microbes from both human and non-human origins, providing a unique venue for microbial interactions, including horizontal gene transfer. Furthermore, stressors present in the built environment could favor the exchange of genetic material in general and the retention of antibiotic resistance genes in particular. Intrinsic and acquired antibiotic resistance both pose a potential threat to human health; these phenomena need to be considered and controlled separately. The presence of both environmental and human-associated microbes, along with their associated antibiotic resistance genes, in the face of stressors, including antimicrobial chemicals, creates a unique opportunity for the undesirable spread of antibiotic resistance. In this review, we summarize studies and findings related to various interactions between human-associated bacteria, environmental bacteria, and built environment conditions, and particularly their relation to antibiotic resistance, aiming to guide “healthy” building design.

## Introduction

The built environment microbiome can be defined as the group of microorganisms, including bacteria, viruses, and eukaryotes (such as yeasts, fungi, and protists) present in any human-constructed environments. Currently in developed countries, exposure to microbes is most likely going to take place in built environments, as humans in modern societies spend nearly 90% of their lifetime indoors [1]. Given the importance of indoor air and built microenvironments on individual exposures [2], the National Academy of Sciences, Engineering, and Medicine recently outlined a research agenda to investigate “the formation and function of microbial communities in built environments and the impacts of such microbial communities on human health” [3, 4]. Therefore, understanding which microbial exposures take place within the built environment and how is of primary importance to thoroughly assess risks for human health.

Microorganisms, and particularly bacteria, have long been primarily associated with infectious disease. Human exposure to microbes can have important negative effects on human health (e.g., infections, including nosocomial infections; allergies; inflammation). Since the first discovery of penicillin in 1928 [5] and its successful use in clinical treatment in 1940 [6], many life-saving antibiotics have been synthesized. However, as early as 1942, four *Staphylococcus aureus* strains were found to be resistant to penicillin in hospitalized patients [7]. Nowadays, the World Health Organization lists antibiotic resistance as one of the greatest threats to not only global health, but also food security and development (https://www.who.int/en/news-room/fact-sheets/detail/antimicrobial-resistance). Like bacteria in any environment, indoor microbial communities contain a diverse array of antibiotic resistance genes. Given the proportion of time people spend indoors and the high potential for exposure, the incidence of antibiotic resistance within the built environment microbiome deserves careful scrutiny. Therefore, in this review, we will summarize major findings in indoor microbial ecology, evolution, and the risk of antibiotic resistance in the context of the built environment, with the aim of providing better guidance for future building design.

## The built environment microbiome: general trends and features

Bacteria found in buildings originate from different sources, including soil, plants, water, household insects, pets (if present), outdoor air but also and often humans, in particular bacteria from human skin, feces, and oral cavity [7–9]. To date, investigated built environments include various buildings, water systems and vehicles, among other microenvironments. A summary of the bacterial taxa most commonly found in the built environment and their variation in different indoor samples from different studies is provided in Table 1. The variation in relative abundances indicates that microbial communities sampled from different indoor environments are likely to be highly heterogeneous. This is consistent with a recent study on dust samples from athletic facilities, where only 26% of the identified bacteria species (*n* = 370) were found in more than half of the buildings. Indoor microbial communities were shown to be highly specific to individual indoor environments, thus the built environment may lack a shared core microbiome [10]. However, one major finding that holds true for all indoor environments is that which specific microbiota are observed in a given location is likely explained by human occupancy of buildings. As an example, a study by Lax et al. [7] confirmed that surface microbial communities tend to be very specific to the home from which they are collected and that the home microbiome reflects the microbiome of its inhabitants. The authors also showed that when the occupants leave a home for a few days, microbial communities living on surfaces in the home decline before increasing again upon the occupants’ return. Humans release their own specific microbes in the space they occupy and that human microbial signature can be used to identify individual occupants [11]. Through the use of source-tracking software, several studies [9, 10, 12] have shown that microbes associated with humans (and pets if any) dominate indoor microbial communities.

**Table 1:** Representative bacterial taxa detected in different indoor environments and surfaces from different studies

Bacterial phylum	Bacterial taxa	Putative origin in the study	Relative abundance in the sample (%)	Type of sample	Study
Proteobacteria	Rhizobiales	Soil	5	Indoor air of family residences	Emerson et al. [106]
	Burkholderiales	Soil	6	Indoor air of family residences	
	Pseudomonadales	Soil, human skin	8	Indoor air of family residences	
	Pasteurellales	Human skin	0.2	Indoor air of family residences	
	Sphingomonadales	Soil, human skin	3	Indoor air of family residences	
	Enterobacterales	Soil, human skin	1	Indoor air of family residences	
Firmicutes	Bacillales	Soil, human skin	12	Indoor air of family residences	
	Clostridiales	Soil, human skin	3	Indoor air of family residences	
Actinobacteria	Actinomycetales	Soil, human skin	20	Indoor air of family residences	
Bacteroidetes	Cytophagales	Soil	1	Indoor air of family residences	
Proteobacteria	Alphaproteobacteria	Human skin	15	Indoor air of a daycare center	Prussin II et al. [117]
	Betaproteobacteria	Human skin	7	Indoor air of a daycare center	
	Gammaproteobacteria	Human skin	30	Indoor air of a daycare center	
Firmicutes	Bacilli	Human skin	10	Indoor air of a daycare center	
	Clostridia	Human skin	4	Indoor air of a daycare center	
Actinobacteria	Actinobacteria	Human skin	10	Indoor air of a daycare center	
	Thermoleophilia	Human skin	1.5	Indoor air of a daycare center	
Bacteroidetes	Flavobacteriia	Human skin	3	Indoor air of a daycare center	
	Bacteroidia	Human skin	1.5	Indoor air of a daycare center	
	Saprospirae	Human skin	1.5	Indoor air of a daycare center	
Proteobacteria	Alphaproteobacteria	Human skin, pets, house surfaces	5	Swabs from human skin, pets, house surfaces	Lax et al. [7]
	Betaproteobacteria	Human skin, pets, house surfaces	8	Swabs from human skin, pets, house surfaces	
	Gammaproteobacteria	Human skin, pets, house surfaces	16	Swabs from human skin, pets, house surfaces	
Firmicutes	Bacilli	Human skin, pets, house surfaces	27	Swabs from human skin, pets, house surfaces	
	Clostridia	Human skin, pets, house surfaces	8	Swabs from human skin, pets, house surfaces	
Actinobacteria	Actinobacteria	Human skin, pets, house surfaces	5	Swabs from human skin, pets, house surfaces	
Bacteroidetes	Flavobacteriia	Human skin, pets, house surfaces	3	Swabs from human skin, pets, house surfaces	
	Bacteroidia	Human skin, pets, house surfaces	1.5	Swabs from human skin, pets, house surfaces	
	Sphingobacteria	Human skin, pets, house surfaces	1.5	Swabs from human skin, pets, house surfaces	
Proteobacteria	Proteobacteria	Refrigerator	62	Swabs of refrigerator surfaces in Korea	Jeon et al. [118]
Proteobacteria	Proteobacteria	Toilet	16	Swabs of toilet seat surfaces in Korea	
Firmicutes	Firmicutes	Refrigerator	10	Swabs of refrigerator surfaces in Korea	
Firmicutes	Firmicutes	Toilet	11	Swabs of toilet seat surfaces in Korea	
Actinobacteria	Actinobacteria	Refrigerator	41	Swabs of refrigerator surfaces in Korea	
Actinobacteria	Actinobacteria	Toilet	43	Swabs of toilet seat surfaces in Korea	
Bacteroidetes	Bacteroidetes	Refrigerator	10	Swabs of refrigerator surfaces in Korea	
Bacteroidetes	Bacteroidetes	Toilet	9	Swabs of toilet seat surfaces in Korea	
Proteobacteria	Alphaproteobacteria	Cutting board	13.7	Swabs of kitchen cutting board surface	Dunn et al. [119]
		Kitchen counter	13.9	Swabs of kitchen counter surface	
		Refrigerator	8.7	Swabs of the surface of a refrigerator	
		Toilet seat	2.1	Swabs of toilet seat surface	
		Pillowcase	4.7	Swabs of pillowcase surface	
		Door handle	13.9	Swabs of main door exterior handle surface	
		Television	15.2	Swabs of the television screen surface	
		Door trim (interior)	21.7	Swabs of the upper door trim on the outside surface of an exterior door	
		Door trim (exterior)	30	Swabs of the upper door trim of an interior door surface	
	Betaproteobacteria	Cutting board	4.7	Swabs of kitchen cutting board surface	
		Kitchen counter	3.7	Swabs of kitchen counter surface	
		Refrigerator	3.3	Swabs of the surface of a refrigerator	
		Toilet seat	1.7	Swabs of toilet seat surface	
		Pillowcase	7.8	Swabs of pillowcase surface	
		Door handle	9	Swabs of main door exterior handle surface	
		Television	7.5	Swabs of the television screen surface	
		Door trim (interior)	7.5	Swabs of the upper door trim on the outside surface of an exterior door	
		Door trim (exterior)	7	Swabs of the upper door trim of an interior door surface	
	Gammaproteobacteria	Cutting board	18.1	Swabs of kitchen cutting board surface	
		Kitchen counter	8.4	Swabs of kitchen counter surface	
		Refrigerator	8.4	Swabs of the surface of a refrigerator	
		Toilet seat	2	Swabs of toilet seat surface	
		Pillowcase	8	Swabs of pillowcase surface	
		Door handle	3.7	Swabs of main door exterior handle surface	
		Television	6.2	Swabs of the television screen surface	
		Door trim (interior)	5.1	Swabs of the upper door trim on the outside surface of an exterior door	
		Door trim (exterior)	8.2	Swabs of the upper door trim of an interior door surface	
Firmicutes	Bacilli	Cutting board	25.6	Swabs of kitchen cutting board surface	
		Kitchen counter	34.8	Swabs of kitchen counter surface	
		Refrigerator	48	Swabs of the surface of a refrigerator	
		Toilet seat	23.7	Swabs of toilet seat surface	
		Pillowcase	23.6	Swabs of pillowcase surface	
		Door handle	18.6	Swabs of main door exterior handle surface	
		Television	17.5	Swabs of the television screen surface	
		Door trim (interior)	12	Swabs of the upper door trim on the outside surface of an exterior door	
		Door trim (exterior)	6.5	Swabs of the upper door trim of an interior door surface	
	Clostridia	Cutting board	2.6	Swabs of kitchen cutting board surface	
		Kitchen counter	2.9	Swabs of kitchen counter surface	
		Refrigerator	2.2	Swabs of the surface of a refrigerator	
		Toilet seat	32.2	Swabs of toilet seat surface	
		Pillowcase	11.8	Swabs of pillowcase surface	
		Door handle	6.6	Swabs of main door exterior handle surface	
		Television	6.1	Swabs of the television screen surface	
		Door trim (interior)	3.9	Swabs of the upper door trim on the outside surface of an exterior door	
		Door trim (exterior)	2	Swabs of the upper door trim of an interior door surface	
Bacteroidetes	Bacteroidia	Cutting board	1.1	Swabs of kitchen cutting board surface	
		Kitchen counter	1.3	Swabs of kitchen counter surface	
		Refrigerator	1	Swabs of the surface of a refrigerator	
		Toilet seat	7.8	Swabs of toilet seat surface	
		Pillowcase	9.4	Swabs of pillowcase surface	
		Door handle	4.3	Swabs of main door exterior handle surface	
		Television	4.5	Swabs of the television screen surface	
		Door trim (interior)	2.5	Swabs of the upper door trim on the outside surface of an exterior door	
		Door trim (exterior)	0.6	Swabs of the upper door trim of an interior door surface	
	Sphingobacteria	Cutting board	1.7	Swabs of kitchen cutting board surface	
		Kitchen counter	2.5	Swabs of kitchen counter surface	
		Refrigerator	2.5	Swabs of the surface of a refrigerator	
		Toilet seat	0.3	Swabs of toilet seat surface	
		Pillowcase	1.2	Swabs of pillowcase surface	
		Door handle	3.4	Swabs of main door exterior handle surface	
		Television	4.5	Swabs of the television screen surface	
		Door trim (interior)	4.9	Swabs of the upper door trim on the outside surface of an exterior door	
		Door trim (exterior)	4.7	Swabs of the upper door trim of an interior door surface	
Actinobacteria	Actinobacteria	Cutting board	21.3	Swabs of kitchen cutting board surface	
		Kitchen counter	16	Swabs of kitchen counter surface	
		Refrigerator	11.6	Swabs of the surface of a refrigerator	
		Toilet seat	24.5	Swabs of toilet seat surface	
		Pillowcase	18.6	Swabs of pillowcase surface	
		Door handle	19.5	Swabs of main door exterior handle surface	
		Television	18.9	Swabs of the television screen surface	
		Door trim (interior)	18.2	Swabs of the upper door trim on the outside surface of an exterior door	
		Door trim (exterior)	13.4	Swabs of the upper door trim of an interior door surface	
Acidobacteria	Acidobacteria	Cutting board	0.4	Swabs of kitchen cutting board surface	
		Kitchen counter	0.8	Swabs of kitchen counter surface	
		Refrigerator	0.2	Swabs of the surface of a refrigerator	
		Toilet seat	0.2	Swabs of toilet seat surface	
		Pillowcase	0.5	Swabs of pillowcase surface	
		Door handle	1	Swabs of main door exterior handle surface	
		Television	1.1	Swabs of the television screen surface	
		Door trim (interior)	1.5	Swabs of the upper door trim on the outside surface of an exterior door	
		Door trim (exterior)	4.7	Swabs of the upper door trim of an interior door surface	

For each taxon, the relative abundance in the collected sample, the putative origin in the study, and the type of sample in which the taxon was detected are described. References of each study are listed in the last column of the table

Moreover, geographical patterns in indoor microbiomes have been observed [13] in a comparison of several ribosomal RNA gene-based studies of the microbiome of the built environment. This meta-analysis highlighted the presence of patterns in indoor bacterial communities depending on the geographical location of the buildings and building type. The authors also reported that in all individual studies included in their study, human skin and outdoor air are consistent sources for indoor microorganisms, despite any differences in sampling techniques and experimental protocols. Apart from these observations, global patterns in indoor microbiomes are difficult to discern in part because indoor microbiomes are relatively diverse and heterogeneous. As a reflection of the variety of conditions and characteristics encountered in indoor built environment, the indoor microbiome is often found to be specific to each indoor space. For example, Rintala et al. [14] showed microbial communities in settled dust from two different buildings in Finland differ from each other and the difference between buildings is larger than the difference observed during seasonal variations within a single building. Similarly, in a study examining surfaces in offices located in New York, San Francisco and Tucson, Hewitt et al. [15] found the microbial communities in different locations to be very different from each other despite similarities in surface material and space type.

**Fig. 1:**
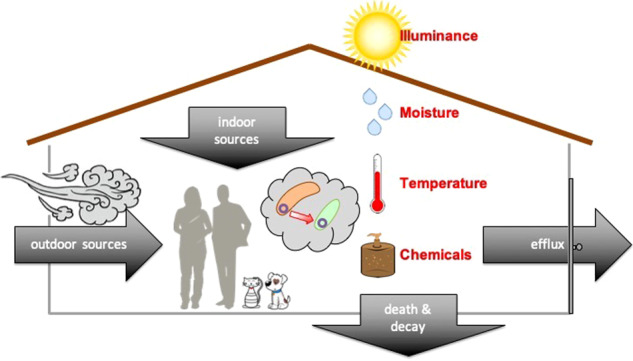
Bacteria from indoor and outdoor sources encounter various stressors in the built environment. The flux of bacteria in the built environment (black arrows), includes sources such as humans (and pets if any) and outdoor air or outdoor environments. While in the built environment, these bacteria from different origins may experience specific selective pressures or stressors (red), including exposure to UV light or luminance in general, low humidity, temperature variation, and the presence of various chemicals such as antimicrobials. Exposure to these stressors may induce the transfer of mobile genetic elements (center)

**Fig. 2:**
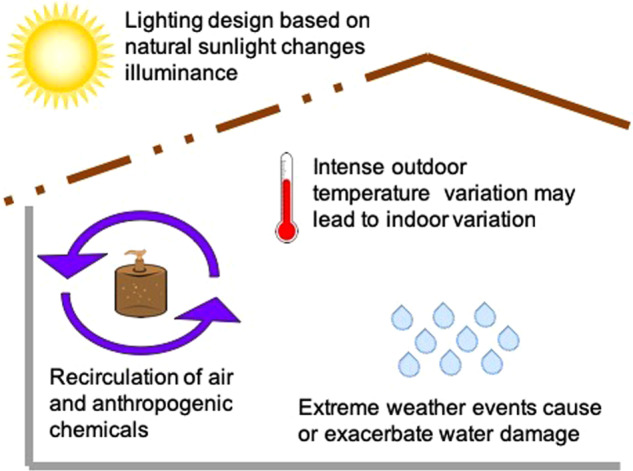
Climate change has implications for indoor environmental quality, including the indoor microbiome. Climate change has prompted innovations in building design and operation to increase energy efficiency. These innovations, such as increased natural lighting and tightened building envelopes may change the conditions experienced by microbes, e.g., illuminance and exposure to chemical stressors. At the same time, changing climate conditions are causing an increase in extreme weather events, leading to increased moisture damage in buildings. Buildings without adequate temperature control may also be subject to more intense variations in indoor temperatures

In addition to human occupancy and geographical location, factors such as humidity, temperature, pH, and the presence of openings allowing infiltration of outdoor air, air filtration, chemical residues, and room occupancy may influence the microbiome of the indoor built environment [16] (Fig. 1). However, the effect of these environmental conditions is small. Temperature, humidity, and illuminance have the most significant, but still very limited, influence on the indoor microbiome [7, 17, 18]. Moreover, the frequency, amplitude and time of exposure to these environmental factors can vary substantially, and some factors are intrinsically related to others. For example, variations in ventilation strategy (i.e., mechanical vs. natural) can directly affect the composition of the indoor microbiome [19], and they could also have an indirect impact by diluting chemicals (e.g., antimicrobials) bacteria are exposed to. Chemicals in the built environment are common. In indoor dust, chemical residues commonly detected include antimicrobials such as triclosan, triclocarban, and parabens. These latter are added to multiple personal care products and home furnishings to avoid mold and bacterial growth [20, 21]. A recent study [22] demonstrated the ubiquity of antimicrobials within the built environment by measuring parabens (methyl-, propyl-, ethyl-, butyl-, and benzyl-paraben), triclosan, and triclocarban in 80 U.S. dust samples collected in homes and athletic facilities. Significant auto-correlation was observed between concentrations of the different parabens, likely reflecting the fact that they are frequently used in combination in the same products and thus have a shared source. In the context of global warming, some additional factors can add to this indoor chemical pollution, such as air pollution, and building engineering is further needed to compensate, mitigate and limit these effects (Fig. 2). Thus, the way we design and operate the buildings in which we spend our time has changed the microbial entities to which humans are exposed [23], as well as the chemical exposures experienced by both human and microbial occupants [24]. In return, humans by their presence indoors also influence the built environment microbiome, each individual’s microbiome specifically participating in the built environment microbiome and leaving a microbial fingerprint after occupying a new place for only a few hours or days [7, 11].

## The built environment: a unique setting for microbial interaction and evolution

The built environment is a place where “environmental bacteria” originating from different outdoor environmental niches such as soil, air, water, as well as those associated with non-human hosts (i.e., plants and animals), mix with human-associated bacteria (Fig. 1). While the indoor microbiome is heavily influenced by the occupants’ microbiomes, there are still many environmental and non-human host-associated microbes. Indoor environments thus represent a unique meeting ground for microbial passengers from various origins.

Bacteria encounter various stresses in their natural environments and respond defensively (e.g., sporulation, biofilm formation, cannibalism, etc…) [25]. Many bacterial stress responses enable them to survive unfavorable and changing conditions, and can also confer resistance to antibiotics [26]. Environments, such as sewage and greywater, also harbor microbial communities from diverse origins, including human and non-human-associated microbes. In sewage and greywater, water and nutrients such as carbon are present in abundance. Thus, the main sources of stress for bacteria in these environments are likely to be from chemicals, like antimicrobials, antibiotics, pesticides, herbicides, and heavy metals at lower concentrations. Consequently, bacterial communities in these types of environments become structured based on the ability of individual members or consortia to tolerate the presence of such chemical stressors. In agreement with this hypothesis, a recent study on the sewage microbiomes of 71 different US cities showed that the variability of sewage microbial communities between different US populations was lower than the interpersonal variation of gut microbiomes [27]. Given the mixed nature of this aquatic environment, the probability of interaction between bacterial cells that were originally distant is high [28, 29], allowing for syntrophic interactions, heterogenous biofilm formation, and the exchange of genetic material. All of these strategies can contribute to the structure, resistance, and resilience of bacterial communities.

**Fig. 3:**
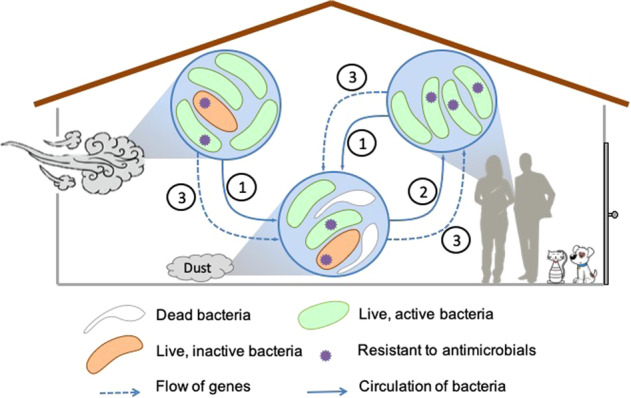
The circulation of bacteria, genes, and antibiotic resistance in the built environment. Indoor bacteria come from both human and environmental sources (1). Once deposited in the indoor environment, there is potential for human exposure (2). In the built environment, mobile genetic elements can be transferred from environmental to host-associated bacteria and vice versa; genetic material can also be taken up by human-associated bacteria upon contact with the indoor microbiome (3). Antimicrobial resistance can be transferred between viable, active bacteria (green) or from nonviable bacteria (white) to viable, inactive bacteria. Viable, inactive bacteria are unlikely to participate in the transfer of mobile genetic elements but may nevertheless be phenotypically resistant to antimicrobials

Similarly, the built environment constitutes a mix of human-associated microbes and “environmental” microbes. However, in contrast to aqueous systems, built environment microbial assemblages are less concentrated. The bacterial density can also highly differ between dust (10^2^–10^7^cells/g) and sludge (10^5^–10^7^cells/g) [25, 30]. Interactions between originally distant cells are thus expected to be more stochastic than in a well-mixed aqueous environment. Nevertheless, the indoor environment is likely to foster interactions between bacteria from different niches through, for example, cooperation via the exchange of genetic material and transfer of mobile genetic elements (Fig. 3); syntrophy with production of secondary metabolites; cell-cell communication through the production of signaling molecules; cannibalism in sporulating bacteria [25] or recycling of dead bacteria by living bacteria. Currently, little information is available on the nature of interactions between bacteria originating from humans and those from soil.

While microbes of different origins are often observed to co-occur in the built environment [8, 10], most of these observations do not distinguish between viable cells and necromass [31], which could be important for certain interactions. These interactions between human- and soil-associated bacteria are especially pertinent in case of exposure to anthropogenic chemicals, which are a common selective pressure in the built environment. Assuming viable bacterial cells are present and surviving in a specific built environment, exposure of bacteria to chemicals, especially antimicrobial chemicals, could lead to the dissemination of antibiotic resistance genes through the transfer of mobile genetic elements between soil- and human-associated bacteria. In addition, metabolically active bacteria could be a source of antibiotics, expressed to outcompete other bacteria in a shared resource-limited niche [32–34].

Another important yet largely unexplored mechanism for interaction between bacteria is the presence of bacteriophage viruses. A prior study [35] reported that viruses prefer low humidity compared to bacteria and show higher abundance compared to bacteria below 40% relative humidity. Taking into account how viruses shape bacterial communities, both through predation and as a mechanism of genetic exchange, viruses are of primary interest to better assess indoor microbiomes. It is particularly important as viruses can participate to the dissemination of genes, including antibiotic resistance genes, between bacteria and can thus participate in the long-term adaptation of the indoor bacterial community to indoor stresses.

The built environment has extreme stressors for bacteria, coupled with unusual patterns of dispersal. With its variability of conditions and the diverse origins of its bacterial inhabitants, the built environment thus constitutes a unique model to study mechanisms of bacterial colonization, interaction and evolution under resource limitation and desiccation.

## Stress response and antibiotic resistance

Sources of stress for bacteria in the built environment are multiple. Some common strategies for bacteria to resist physical or nutrient-related stresses confer temporary resistance to antibiotics and antimicrobials, when that phenotype is expressed. For example, sporulation in response to temperature shock or desiccation may also inhibit the effect of antimicrobials on spores, as is the case with benzalkonium chloride [36]. This antimicrobial is increasingly used in personal care products, as well as paints and is known to be ineffective against spores [37]. Other examples of resistance phenotypes include dormancy, production of osmoprotectants, biofilm formation, thickening of the cell membrane and cell wall, increased expression of efflux pumps, and decreased expression of porins in the membrane to reduce permeability [38–43]. Genes encoding these functions are typically located on the chromosome and thus can be considered intrinsic. However, they are not constitutively expressed, nor are they necessarily considered antibiotic resistance genes.

Another mechanism of resistance to stress for bacteria in the built environment includes resistance to antibiotics specifically. Like bacteria in any environment, indoor microbial communities contain a diverse array of antibiotic resistance genes [44]. Antibiotic resistance is an ancestral mechanism of competition between bacteria living in the same niche, especially in resource-limited environments. Antibiotic resistance genes can thus be found in all environments, and were part of bacterial gene arsenals well before the extensive use of synthetic antibiotics by humans in clinical, agricultural, and veterinary settings [45]. The use of antibiotics by humans has, however, triggered a change in the ecological function of antibiotic resistance genes and their acquisition by pathogenic bacteria [46]. Because of the ubiquity of antibiotic resistance genes in all environments, and the variety of possible functions encoded by even fairly homologous genes, it is difficult to define antibiotic resistance in environmental strains and to compare this resistance to bacterial isolates from clinical settings [47]. In this context and in the era of a predominant use of molecular tools for the investigation of the built environment microbiome where both environmental and human-associated bacteria coexist, it is imperative to clearly distinguish between antibiotic resistance genes that are part of the classic resistance genotype and phenotype of a given microorganism—the “intrinsic” resistome—from antibiotic resistance genes that are acquired through transfer of mobile genetic elements, such as transposons, integrons, plasmids, or viruses—the “acquired” resistome.

Intrinsic antibiotic resistance in bacteria is generally caused by three main phenomena: lack/loss of target for antibiotics, presence of a chromosomally encoded enzyme mediating antibiotic resistance, and reduction of membrane permeability and activation of efflux pumps to prevent or limit antibiotic influx into the bacterial cell [48]. Intrinsic resistance is automatically transferred by replication of the chromosome during cell division from a given bacterium to its daughter cells. Intrinsic resistance is also thought to be constitutively expressed, which has consequences in terms of adaptation of bacteria to built environment stressors, as constitutive expression may incur a fitness cost. Nevertheless, this cost may be justified, e.g., in the presence of sporadic pulses of antimicrobial chemicals.

**Fig. 4:**
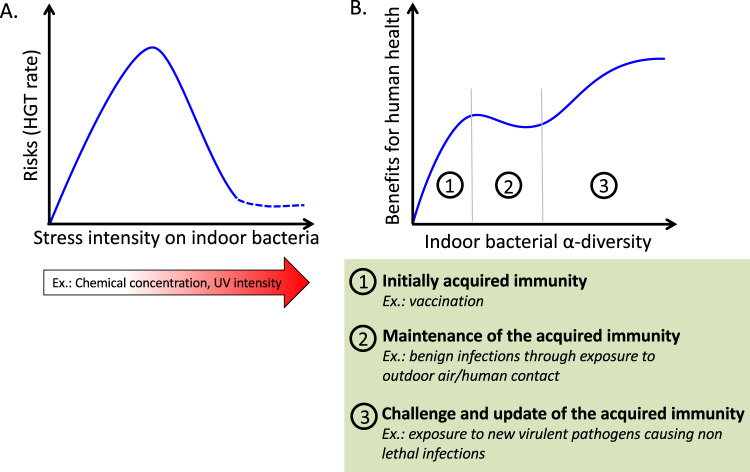
Stress may increase the human health risk posed by bacteria, but exposure to a diverse microbiome likely confers benefits. **a** Many stresses can induce horizontal gene transfer in bacteria, potentially increasing the spread of antibiotic resistance even when those genes do not confer a direct benefit. However, horizontal gene transfer can only occur until the intensity of the stress becomes high enough to inhibit bacterial survival. **b** A previously unexposed human (i.e., neonate) benefits rapidly from a high diversity of microbial exposures, as it allows acquired immunity to develop. Regular exposure to a relatively high-bacterial alpha-diversity helps maintain the acquired immunity over time. However, continued exposure to a high diversity of bacteria increases the chance of exposure to pathogens

In order to evaluate the interest of using intrinsic vs. acquired resistance resistance genes in the presence of a chemical stressor, the concentration of the chemical and its properties of diffusion and dispersion need to be taken into consideration. In the built environment where chemicals are ubiquitous, the fate of chemicals can be modeled by a hyperbolic curve with input concentrations rapidly decreasing due to diffusion and dispersion [49, 50]. The fate of any particular chemical depends on properties of the chemical itself, such as volatility and stability. In addition, properties of the particular built environment will also come into play, e.g., design and operation of the ventilation system, and the surface properties of materials present in the building governing sorption. When the chemical has just been introduced to a surface or in a room, the bacteria are exposed to a high concentration, requiring an instantaneous response from the bacteria to counteract the stress. Constitutively expressed, chromosomally encoded resistance strategies are best to provide an immediate response to the presence of a chemical stressor at high concentration. In this case, the immediate appearance of a high concentration of the chemical could select for specific bacteria that are already able to cope with this or similar stresses. However, constitutive expression of multiple resistance genes, especially in an environment where resources are limited, can constitute a burden for bacteria (Fig. 4a).

It is worth noting, however, that the presence of intrinsic antibiotic resistance genes is not necessarily connected to the presence of a stressor, as not all genes that we attribute to intrinsic antibiotic resistance in fact contribute to resistance and protection of a microorganism in nature [46]. Even for those that do, antibiotic resistance may not be their sole purpose. Some of these genes are primarily involved in other cellular functions and thus their constitutive expression is necessary, regardless of the presence of chemical stressors. One example of a necessary “resistance” gene is multidrug efflux pumps [38]. Multidrug efflux pumps are mostly located on chromosomes, and only a few are found in association with a mobile genetic element [51–57]. Multidrug drug efflux pumps play important roles in the physiology of bacteria, including maintenance of the cell shape, uptake of extracellular nutrients, biofilm formation, signal transduction and regulation of osmosis at the membrane and cell wall [38, 58, 59]. All of these functions are necessary to the cell, even in the absence of toxic compounds. The usefulness of these genes in different environmental contexts, whether stressful or not, decreases the fitness cost for the bacteria, favoring the continued maintenance of these genes. Furthermore, some putative multidrug efflux pumps do not in fact confer resistance to antibiotics unless they have a specific mutation, e.g., the *oprM* gene in *Pseudomonas aeruginosa* [60–64]. Thus, supposedly costly constitutively expressed intrinsic antibiotic resistance genes may be extremely beneficial to bacteria, even (or especially) in the nutrient- and water-limited built environment.

Regardless of fitness costs or levels of expression, intrinsic antibiotic resistance are chromosomally located and thus unlikely to be gained, lost, or transferred. This is in contrast to acquired antibiotic resistance, which is associated with mobile genetic elements. In the case of acquired resistance, the genes in question can be transferred to daughter bacteria but can also be lost if the pressure originally triggering the transfer and acquisition of the genes drops below the selective threshold. Acquisition of new exogenous genes is likely to occur when the concentration of a chemical stressor in the built environment starts to fade and reaches a sublethal but still stressful level. The acquisition of new genes through mobile genetic elements, particularly plasmids, imposes a biosynthetic burden on bacteria [65]. Acquired genes thus must confer a clear advantage for the bacteria under stress.

Despite the fitness burden that might be associated with acquired antibiotic resistance, acquiring plasmids still confers two main advantages. First, it is possible in some cases for the bacteria to develop a more intense response to a stressor due to the higher copy number of specific plasmid-based resistance genes [66]. Second, the bacteria can decrease the cost of maintenance of plasmid-based genes through the loss of the plasmid or down regulation of gene expression in the absence of a stressor, allowing higher flexibility in stress response [67]. Given these advantages, under a sublethal but stressful concentration of a chemical and under dry and nutrient-limited conditions, the acquisition of exogenous resistance genes could constitute an efficient response. Retention of the acquired genes can be temporary, e.g., if the chemical stressor disappears. However, if the plasmid-based genes also confer other advantages, such as in nutrient uptake, thus compensating for the fitness cost of plasmid acquisition and maintenance, the plasmid could be retained indefinitely [68–70]. If the acquired plasmid is stable in the bacterial host, it can be transferred to other bacteria, including members of different genera, thereby helping maintain diversity in a stressed microbial community [71]. The presence of persistent organic antimicrobials in the built environment, either embedded in antimicrobial surfaces or accumulated in dust, may favor the prolonged retention of mobile genetic elements such as plasmids [72, 73].

Horizontal gene transfer and acquire resistance genes can play a key role in the development and maintenance of diversity and resilience in a microbial community. A recent study from Ellison et al. [74] showed for the first time that *Vibrio cholerae* can append a competence pilus to mediate exogenous DNA uptake, which is the first step for natural transformation, from nonviable cells. This phenomenon might be of particular importance in the built environment microbiome, as this environment is hypothesized to be a “microbial wasteland” mainly populated by nonviable bacteria [75]. While nonviable bacteria are incapable of metabolic activity or reproduction, they constitute a tremendous reservoir for exogenous genes, including genes conferring antibiotic resistance. The process of natural transformation thus likely plays a critical role for bacterial survival in dry, nutrient-limiting built environments, particularly in the presence of antimicrobials or other chemical stressors. This process is especially interesting as the nature of the media and substrates present in the built environment precludes bacterial dissemination via motility, as opposed to aqueous environments that permit chemotaxis. However, there is currently insufficient evidence to confirm or refute these hypotheses.

Acquired antibiotic resistance gene dynamics in the built environment could also reveal recent and ongoing selective pressures experienced by the indoor microbial community. The types of resistance genes contained within the community might vary according to the time and intensity of the exposure to the selective pressure, e.g., the presence of antimicrobials in indoor dust. If the concentration of the antimicrobial is very high and was introduced suddenly, the microbial community may be biased toward the selection of intrinsically resistant bacteria. However, if the antimicrobial were introduced or removed gradually, the community might favor a higher rate of gene transfer between members of a complex microbial community, allowing for acquisition of new antimicrobial resistance genes.

Ultimately, most likely both intrinsic and extrinsic strategies happen concomitantly depending on the fitness cost of plasmid transfer compared to the fitness cost of chromosomal resistance [66]. However, the extent to which each strategy dominates in different complex bacterial communities and under different conditions in the built environment remains an open and unexplored question. An estimation of the fitness cost of resistance gene dissemination within the built environment is needed. In the built environment, multiple sources of stress for bacteria can coexist, but many stressors can be altered through building design and operation [76]. For example, exposure to ultraviolet (UV) or visible radiation, which can affect dust microbial community composition [77], can be changed through lighting design, and the presence of antimicrobial chemicals can be altered through the selection of different products and finishes [78].

In summary, time, frequency, and amplitude of exposure to a source of stress likely affect the evolution of antibiotic resistance and dissemination of related genes in the indoor microbiome. Understanding how these stressors affect indoor microbial ecology will allow us to manipulate conditions to disfavor scenarios that select for highly antibiotic-resistant “superbugs.”

## Understanding the dissemination of antibiotic resistance in the built environment

The use of molecular and culture-independent tools has unveiled a new dimension of the bacterial diversity within inhabited buildings. Complementing next-generation DNA sequencing with orthogonal molecular or culture-based methods will further allow us to distinguish between live and dead cells. Better characterization of metabolic activity can be achieved through the use of metabolomics and proteomics [16].

The assessment of the viability and activity of the bacteria detected within the built environment is crucial to provide an accurate assessment of the risk related to antibiotic resistance genes, which is currently regarded as one of the top threats to public health worldwide [79, 80]. Chemical stressors will specifically affect viable bacteria, which are the only cells able to integrate and transfer genes to other cells, as opposed to nonviable cells, which nevertheless constitute a reservoir of genes (Fig. 3). However, the sole presence of a chemical does not mean systematic horizontal gene transfer as the presence of a chemical stressor can elicit other specific response mechanisms, e.g., the modification of a drug target through mutation in a gene or a general stress response. Interestingly, it has been reported that stressors such as halogenated organic chemicals (e.g., antimicrobials), metals or nanoparticles, are likely to incite horizontal gene transfer and dissemination of resistance to multiple chemicals, including antibiotics, through mobile genetic elements [81, 82]. In contrast, some other stressors such as desiccation are unlikely to trigger horizontal gene transfer. The identification of viable taxa in the built environment is essential in order to predict the global bacterial response to specific stressors, and to anticipate the direction of potential horizontal gene transfers and natural transformation phenomena during which free DNA in the environment enters viable organisms [74].

The question of the stability and host range of acquired genes over time is also central in the prediction of the antibiotic resistance dissemination, particularly in mixed communities containing highly diverse environmental and human-associated bacteria. The stability of an acquired antibiotic resistance gene depends on the presence of an external selective pressure supporting the maintenance and dissemination of this antibiotic resistance gene. Genes can be transiently acquired in the built environment but quickly lost in the absence of continued selection. The host range of the mobile genetic element carrying a resistance gene is also important for determining how far it will spread. Host range is particularly critical in the case of plasmids. Although some plasmids have a broad host range and can invade a large fraction of a complex microbial community [71], some plasmids can only be transferred to similar taxa [83, 84]. Even in communities with low diversity, dissemination of plasmids could be limited, as closely related plasmids cannot coexist over time in the same cell, as illustrated by the concept of plasmid incompatibility groups.

In addition to plasmid stability and viability, two phenotypes in particular stand out in terms of their relevance to understanding the dissemination of antibiotic resistance genes: sporulation and biofilm formation. Both phenotypes contribute to antimicrobial resistance but have opposite effects on horizontal gene transfer. Sporulated organisms may still be highly relevant to human health (e.g., *Clostridium difficile*) [85, 86] but are unlikely to participate in horizontal gene transfer, either as donors or recipients, while sporulated in the environment. In contrast, biofilm formation [26] greatly facilitates the transfer of genes, including antibiotic resistance genes, between members of the biofilm [87–89]. Since a significant proportion of bacteria are able to sporulate, and since these bacteria may not be detected through techniques such as high-throughput sequencing due lysis and extraction difficulties [90] or their low DNA content or cell number [91], cultivation remains one of the most accessible approaches to recover this fraction of the microbiome. Nevertheless, it is vital to recognize that many bacterial species, including human pathogens of clinical importance (e.g., *Staphylococcus aureus*, *Vibrio cholera*, *Mycobacterium tuberculosis*, etc.), are capable of entering into a physiological viable but non-culturable (VBNC) state under stressful conditions [92, 93]. Such stress response largely undermines our ability to comprehensively detect pathogens using conventional cultivation-based approaches.

## Risks vs. rewards: the trade-off between infection prevention and immune function

It has long been thought that the presence of bacteria in our surroundings constitutes a threat for human health. That mindset has led to the widespread use of products and technologies to attempt to eliminate bacteria around us. While the adage that the only good microbe is a dead microbe is untrue, some bacteria do present a threat to human health (Fig. 4b). Threats can include frank pathogens but also opportunistic pathogens, non-pathogens carrying mobile antibiotic resistance genes, as well as irritants and allergens. Exposures can take the form of the bacteria themselves, as well as metabolic byproducts [94, 95].

The threat of infection from certain bacteria is genuine; nevertheless, cleaning strategies to remove this threat can defeat their purpose. As an illustration of this concept, consider current strategies implemented in hospitals for terminal room disinfection (disinfection of patient rooms between occupying patients), mainly relying on the use of quaternary ammonium compounds or bleach. Disinfection efficacy is evaluated based on the detection of specific pathogens such as *Clostridium difficile* and other target organisms such as methicillin-resistant *Staphylococcus aureus*, vancomycin-resistant staphylococci and multidrug-resistant *Acinetobacter*. In their recent study, Anderson et al. [86] compared different strategies of terminal room disinfection with the addition of UV-C light and found that the addition of UV light to bleach or quaternary ammonium compounds decreased detection of target organisms but not subsequent *C. difficile* infection. Other recent approaches include engineering surface materials that are “self-decontaminating” from microbes in addition to the application of disinfection products [90]. These approaches are not yet widely applied, and their effectiveness and potential risks are still unknown. The combination of untargeted disinfection methods and targeted evaluation methods leaves a gap in our understanding of how disinfection practices affect indoor microbial ecology as a whole. Furthermore, little insight is available on the long-term selective effect of these strategies, particularly on the hardiest bacteria, like *C. difficile*, and their ability to resist antibiotics and other stressors. Moreover, while extreme disinfection control strategies are needed in specific built environments such as hospitals to protect the health of immune-compromised patients, they may not be needed in other built environments such as the homes of immunocompetent individuals (Fig. 4b). Nevertheless, many chemical and physical disinfectants, including UV sterilization apparatuses, are readily available to consumers.

Chemical disinfectants are targeted at reducing viable biomass on indoor surfaces https://www.ncbi.nlm.nih.gov/pubmed/31429989. However, at present, it is unclear whether it is sufficient to prevent exposure from living bacterial cells, or whether metabolic byproducts, cellular debris, or a combination of the three need to be considered. Furthermore, the routes of exposure, such as ingestion, inhalation, or dermal contact, are still under investigation. While inhalation is plausibly the dominant mechanism [96–99],

it is certainly not the sole route. The role of dermal contact must also be evaluated. Prescott et al. [100] report that human skin microbiota is influenced by the built environment microbiome.

## Conclusions and future directions

The built environment is composed of a wide variety of bacteria originated from both environmental sources and human origins. In order to survive in a dry, nutrient-limiting built environment, particularly in the presence of antimicrobials, many bacteria become resistant through (1) acquisition of antibiotic genes, (2) expression of intrinsic tolerance, and (3) activation of stress response mechanisms that unintentional confer antibiotic resistance. Studies have shown so far that exchanges between humans and the built environment occur. However, many more studies are needed to understand microbial exposure effects on humans and which microbial taxa humans preferentially transfer to the built environment. We have not yet fully quantified these exchanges, established their directionality or been able to design effective and appropriate interventions [101–103]. Efforts to ensure public health within the built environment should continue to impose limitations on microbial growth, but more importantly to regulate and control exposure to specific microbes. Research is necessary to determine how to manage trade-offs between one benefit (i.e., limiting bacterial pathogens) and the other (i.e., promoting harmless or beneficial microbes; Fig. 1).

The high amount of heterogeneity of the built environment, coupled to the low amount of biomass, constitutes a formidable challenge in defining and understanding the indoor microbiome. While the latter challenge will be resolved by technical advances, the former requires more nuanced experimental design. The challenge of heterogeneity calls for more studies of the built environment microbiome through multiple spatial scales from entire buildings, to individual rooms, to surfaces within rooms. Thus far, many studies have been performed at a large scale, comparing, e.g., the microbiomes of several houses located in different geographical areas [103–106] in order to determine geographical patterns. However, few studies tackle the question of how representative a given sample is of a particular building’s microbial community composition or structure. The microbial diversity in the built environment is taxonomically heterogeneous and so is the diversity of ARG encountered in this environment, there has been thus no “core” built environment microbiome identified in the built environment [10]. Since the indoor microbiome is also a function of occupants’ microbiome, both longitudinal and spatial studies of the built environment coupled with characterization of the occupants’ microbiome (e.g., Lax et al. [7]) are necessary. Longitudinal studies will further unveil the biological variability of indoor microbial communities and the dynamics of microbial interactions.

Since the indoor microbiome is also a function of occupants’ microbiome, both longitudinal and spatial studies of the built environment coupled with characterization of the occupants’ microbiome (e.g., Lax et al. [7]) are necessary. Moreover, the analyses of spaces with limited occupant density and diversity compared to public spaces, where the diversity and density of occupants is likely to be higher, over different time and spatial scales could better define the limits of indoor microbiome heterogeneity. This would likely highlight clearer trends and global patterns by essentially averaging the microbial input from different occupants, similar to what has been recently observed in sewage [27, 107, 108]. Studies examining spatial and temporal variation in the indoor microbiome, combined with epidemiological records, will also shed light on relationships between the indoor microbiome, building features, and health outcomes. However more efforts need to be made in order to standardize sampling methods of the indoor microbiome and metadata for meaningful comparison [109].

Assessing the viability of detected bacteria as well as their metabolic activity, ability to transfer mobile genetic elements, and phenotypic state in situ are crucial parameters in the prediction of the potential dissemination pathways of antibiotic resistance genes. These factors can directly affect human exposure to both nominally harmful and beneficial bacteria within the built environment. While orthogonal methods will improve our ability to assess viability and phenotype, DNA-based methods are useful to detect the potential for antibiotic resistance gene dissemination within the built environment because genes contained within dead bacteria can still constitute a reservoir for antibiotic resistance genes for viable bacteria. Public health risk assessment related to antibiotic resistance dissemination in the indoor microbiome needs to account for both viable and nonviable fractions. Observing metabolic activity of viable bacteria in the built environment would reveal which organisms are most susceptible to which disinfection methods. On a more fundamental level, understanding the relationship between especially the moisture in a building and the corresponding metabolic activity would elucidate how bacteria use building materials as a source of energy and which metabolites they produce affect human health [110, 111]. Ultimately, materials used for construction and surface finishes within the built environment could limit growth of nefarious bacteria or even promote growth of beneficial bacterial communities. Characterizing the metabolic activity of bacteria in the built environment would also yield valuable information on the nature of the interactions occurring in the microbial communities [112], hinting at the most plausible species to be involved in horizontal gene transfer. Metabolic profiling of indoor microbial communities in the built environment is thus a key step in the assessment of microbial risks in the built environment.

Stress responses, such as sporulation or dormancy, constitute a generic response to a broad range of stressors, including drugs, other chemicals, changes in temperature, and desiccation. Defining the exposome of the built environment is also a key also the first step towards the design and engineering of “healthier buildings”. However, reaching a “zero exposure” indoor environment is impossible, and attempting to reach this end would likely aggravate threats to human health by selecting for ultra-resistant microbes. Such a goal would also be undesirable, as increasing evidence suggests that microbial exposures are important for a variety of human health concerns, from allergies to mood [113–116]. The built environment is highly heterogeneous, and the wide variety of environmental conditions is reflected in the high heterogeneity of microbial communities both within and between buildings. The presence of both environmental and human-associated microbes, along with their associated antibiotic resistance genes, in the face of stressors, including antimicrobial chemicals, creates a unique opportunity for the undesirable spread of antibiotic resistance. Given the amount of time humans spend indoors the possibility for human exposure to antibiotic-resistant organisms deserves increased attention.
